# High-Throughput Phenotyping of Canopy Cover and Senescence in Maize Field Trials Using Aerial Digital Canopy Imaging

**DOI:** 10.3390/rs10020330

**Published:** 2018-02-23

**Authors:** Richard Makanza, Mainassara Zaman-Allah, Jill E. Cairns, Cosmos Magorokosho, Amsal Tarekegne, Mike Olsen, Boddupalli M. Prasanna

**Affiliations:** 1 International Maize and Wheat Improvement Center (CIMMYT), P.O. Box MP163, Harare, Zimbabwe; rmakanza@live.co.za (R.M.); j.cairns@cgiar.org (J.E.C.); c.magorokosho@cgiar.org (C.M.); a.tarekegne@cgiar.org (A.T.); 2 International Maize and Wheat Improvement Center (CIMMYT), P.O. Box 1041, Nairobi, Kenya; m.olsen@cgiar.org (M.O.); b.m.prasanna@cgiar.org (B.M.P.)

**Keywords:** aerial sensing, maize, crop phenotyping, senescence, imaging

## Abstract

In the crop breeding process, the use of data collection methods that allow reliable assessment of crop adaptation traits, faster and cheaper than those currently in use, can significantly improve resource use efficiency by reducing selection cost and can contribute to increased genetic gain through improved selection efficiency. Current methods to estimate crop growth (ground canopy cover) and leaf senescence are essentially manual and/or by visual scoring, and are therefore often subjective, time consuming, and expensive. Aerial sensing technologies offer radically new perspectives for assessing these traits at low cost, faster, and in a more objective manner. We report the use of an unmanned aerial vehicle (UAV) equipped with an RGB camera for crop cover and canopy senescence assessment in maize field trials. Aerial-imaging-derived data showed a moderately high heritability for both traits with a significant genetic correlation with grain yield. In addition, in some cases, the correlation between the visual assessment (prone to subjectivity) of crop senescence and the senescence index, calculated from aerial imaging data, was significant. We concluded that the UAV-based aerial sensing platforms have great potential for monitoring the dynamics of crop canopy characteristics like crop vigor through ground canopy cover and canopy senescence in breeding trial plots. This is anticipated to assist in improving selection efficiency through higher accuracy and precision, as well as reduced time and cost of data collection.

## 1. Introduction

Accelerated genetic gain in crop improvement is needed to address the ever-increasing global food production demands [1]. In most cases, this has to be achieved with limited or reduced resources dedicated to crop improvement. Therefore, improving resource use efficiency in the breeding process should be given high priority. This can be (i) in the form of reduction of selection costs that would enable an increase in the number of replicates or genotypes in field trials or expand the phenotyping network with the same resources or (ii) through the use of genomics-assisted selection methods [2]. From a phenotyping perspective, the selection cost reduction would include the use of methods of data collection that are reliable, more accurate, and, at the same time, low cost and fast.

Over the last decade, there has been a growing interest in exploring high-throughput imaging techniques to dissect quantitative crop phenotypic traits [3,4]. Visible light imaging has been widely reported among other imaging techniques. It makes use of visible light (400–750 nm) comprising the red, green, and blue bands of the electromagnetic spectrum. Visible band images are normally acquired through silicon sensors (CCD or CMOS) fitted on consumer-grade or conventional digital cameras; providing rapid solutions for plant phenotyping at affordable cost [5]. Canopy architecture information such as canopy cover, senescence, leaf area index, gap fraction, and leaf inclination angles can be easily extracted from visible band images using an appropriate image analysis pipeline.

Computer-assisted imaging systems are becoming available for automated plant phenotyping [6,7] but mostly for indoor or greenhouse applications. Although these automated systems enable the capture of detailed, noninvasive information throughout the plant life cycle, the results from controlled environments are distinct from the actual situations that plants will experience in the field, making it difficult to extrapolate the data to the field where the core of plant breeding work usually takes place [8,9]. Field-based phenotyping is increasingly recognized as the only approach capable of delivering the required throughput and accurate description of trait expression in real-world cropping systems [9].

For field phenotyping, there are a number of platforms that are used with various sensors, including multispectral, hyperspectral, IR, and RGB cameras. They range from simple ground-based platforms i.e., monopods and tripods, to complex unmanned ground vehicles and unmanned aerial vehicles (UAVs) [8]. These platforms are not all suitable for use in large breeding trials that are often composed of many plots, as small as a single row. UAVs have emerged as a promising geospatial data acquisition system for a wide range of applications including field phenotyping [10]. They can be used to acquire images over large landscapes capturing plant characteristics within few minutes. With optimum spatial and spectral resolutions, UAVs can provide spatially and spectrally derived parameters for various purposes including crop condition [11], crop forecasting and yield predictions [12], disease detection and nutrient deficiency [13,14], and photosynthetic pigment content [15–17].

Crop vigor and senescence are physiological processes that characterize plant growth and development phases [18]. Crop vigor is an indicator of robustness and rapidness in the growth of plant canopy architecture, which is primarily influenced by its genetic makeup interacting with the environmental factors. Early crop vigor provides the horsepower that drives plant development and yield while senescence marks the final stage of organ development in plants, characterized by a series of degenerative programmed processes leading to plant death [19–22]. Whilst the process is governed by age of the plant organs, it is also influenced by other internal and environmental signals that are integrated into the age information [21]. During the process of senescence, nutrients are mobilized and moved from sources (leaves), where they are manufactured, to sinks for storage [23]. This is accompanied by yellowing of green parts of the canopy following chlorophyll degeneration. When it takes place prematurely, leaf senescence can negatively affect crop yield by reducing the photosynthetic longevity of leaves, thereby reducing the grain filling window [24]. In a maize plant, both processes are critical as they can directly affect crop productivity.

Breeding trials are often extensive and therefore costly to monitor by conventional means, especially for measurements that are time sensitive like visual scores to assess leaf senescence or crop vigor. For example, in the International Maize and Wheat Improvement Center (CIMMYT) maize breeding program for eastern and southern Africa, over ten thousand measurements of leaf senescence are taken in stage IV trials [25]. This method is not only slow, but also prone to human error. The use of more objective and low-cost methods would enable analysis of time sequence data to assess crop vigor and senescence patterns, providing valuable insight into crop growth, sensitivity to stresses, and grain filling duration. The purpose of this study was to evaluate the suitability of an aerial imaging methodology for estimating crop canopy cover and leaf senescence from small plots in maize field trials.

## 2. Materials and Methods

### 2.1 Experimental Setup

**Figure 1:**
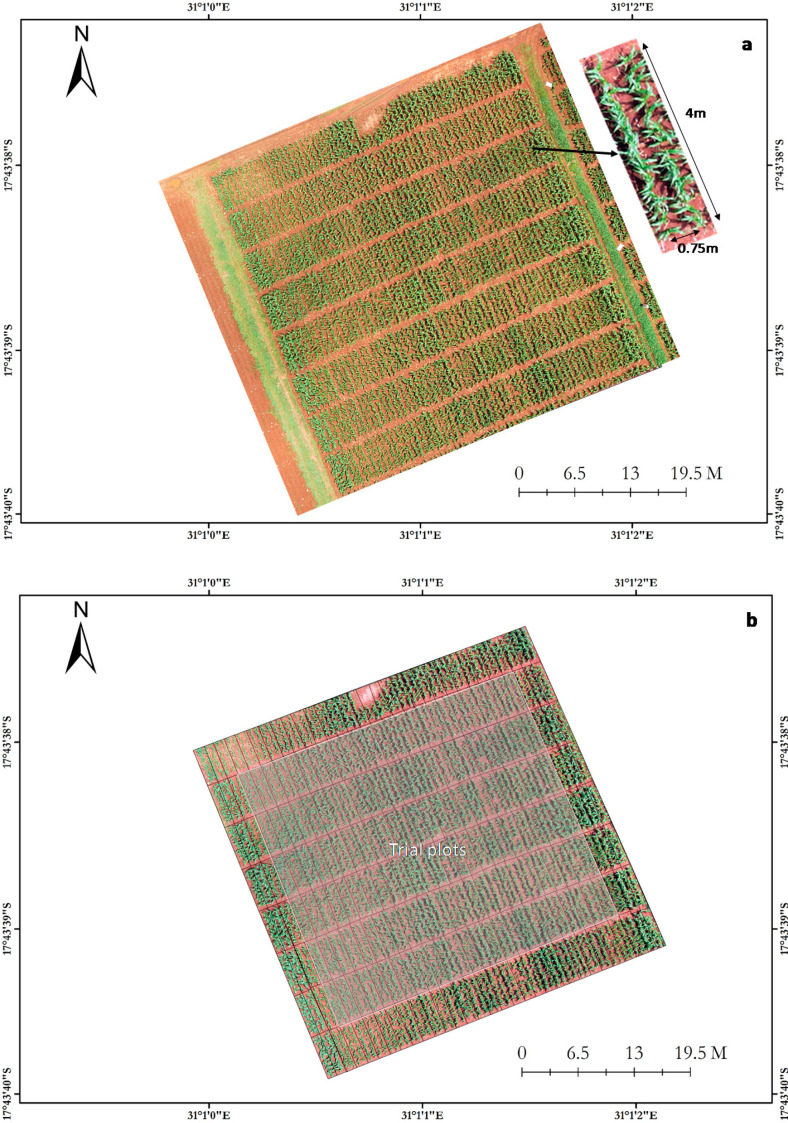
Single-shot aerial image taken from an unmanned aerial vehicle (UAV) platform showing (a) the experimental setup with single plot details and (b) the location of the trial plots.

The study was conducted at CIMMYT research station (17°43ʹ37.21″S, 31°01ʹ00.60″E, and altitude 1489 m above sea level) in Harare, Zimbabwe. Three breeding trials composed of 50 varieties each were planted in three replicates on 3 December 2016 using an alpha lattice design. These varieties were hybrids selected based on their yield potential and stress tolerance. Each variety was represented by 2 row plots (replicated three times) that were 4 m long with inter-row spacing of 0.75 m and in-row spacing of 0.25 m (Figure 1a,b). Therefore, each trial had a total of 150 plots. The three trials were planted on a block that was depleted of nitrogen by removing all crop residues and without any additional nitrogen fertilizer for five consecutive cropping seasons. To ensure a weed-free growing environment for the maize crop, weeds were adequately controlled during the early stages of plant development by using pre-emergence and post-emergence herbicides. In the late vegetative stages, hand weed control was used to remove a few scattered weeds across the trial. In addition, the plant population was also maintained by effective pest management and thinning during early vegetative stages of plant growth.

### 2.2 Image Acquisition

All the flight missions for taking images of the maize field were carried out using a commercially available UAV (Mikrokopter® ARF Okto XL, HiSystems GmbH, Moormerland, Germany). The UAV can carry a maximum payload of approximately 4 kg on board. The UAV was flown at a speed of 8 m/s and a height of 80 m above ground. Images were taken using a Panasonic camera (Lumix DMC GX7) with a resolution of 16 megapixels, fitted underneath the UAV. The camera weighed 512 g, including a battery and a 20 mm fixed focal lens. It was fixed at nadir on a two-axis aluminium–carbon fiber camera mount (servogimbal MK HiSight SLR2) suitable for big cameras which is able to provide them with compensation via the two tilt servos in the pitch and roll directions. This minimized the effect of airframe motion caused by wind. The camera was configured to burst mode at a shooting rate of one image every 5 s and saved in JPEG format on an SDXC (Secure Digital eXtended Capacity) card installed in the camera. A high shutter speed of 1/800 s and a wider aperture of f/5 were used to capture images with minimum blurredness. The vertical and horizontal view angles of the camera were 32° and 46.8°, respectively, and the image size was 3448 × 4592 in pixel dimensions. Therefore, images taken from a height of 80.0 m aboveground capture a 52 m × 69.2 m area (corresponding to 270 plots) with a spatial resolution of 1.51 cm pixel−1. To minimize shadow effects and canopy reflectance, images were taken under diffuse light conditions (cloudy weather or very early in the morning). A total of three flight missions were conducted during the 2015–2016 growing season at 42, 75, and 96 days after sowing (DAS), which coincided with vegetative, post-flowering, and late grain filling stages, respectively.

**Figure 2:**
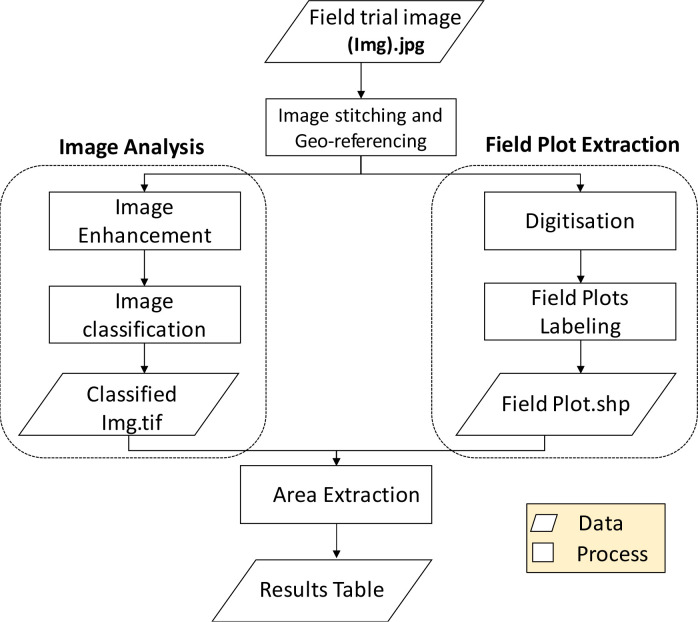
Simplified workflow diagram of the image processing main steps.

Prior to every flight mission, four white wooden boards measuring 30 cm × 20 cm were laid out at the four corners and two at the central edges of the trial field as ground control points (GCPs) and their positions recorded using a 1 cm resolution handheld Geo 7X GPS (Global Positioning System) unit activated for high-accuracy logging via an override tool (Trimble, Sunnyvale, CA, USA). The white color was used to easily distinguish the GCPs from the surrounding vegetation cover in the software during geo-referencing of the images. After every flight, corresponding to each of the three developmental stages that were assessed, raw images stored in the camera memory were transferred to a personal computer (PC) for preprocessing. The images were then imported into ArcGIS 10.1 software for processing. Figure 2 shows a summary of the main image processing steps.

### 2.3 Image Processing

#### 2.3.1 Image Preprocessing

Prior to plot information extraction, AutoPano Pro 4.4 (Kolor SAS, Francin, France; www.kolor.com) software was used to generate mosaics from raw images. The detection control point RANSAC (random sample consensus) model was set to homography (recommended for a changing panorama point of view) and lens distortion correction enabled with multiple viewpoints for optimization. Finally, the projection was set to ortho. The geo-referencing step was performed in ArcGIS 10.1 software by adding six GCPs manually to the mosaiced image using WGS_1984 as a spatial reference. Accurate estimation of the GCPs position was performed by zooming into the image and adding the coordinates. According to GCP positions, the elevation within the field was quite uniform.

#### 2.3.2 Field Plot Extraction

The ArcGIS editor tool was used to create field plots by digitizing them on top of the geo-referenced image mosaics. Field plot boundaries were created manually as polyline features by digitizing the middle of interplots space with the projected coordinates system WGS_1984_UTM_Zone_36S. Compared with polygons, polyline features were more flexible, faster, and easier to use for generating boundaries due to the irregular shapes of field plots as they were hand planted. To generate field plots, polylines were converted to polygons using the Feature to Polygon tool and exported as a shapefile. The conversion step automatically generated the size of each plot as an area (m2) labelled with a unique ObjectID in the attribute table of the field plot file. Other field plot attributes on the trial map, i.e., trial name, plot name, variety name, were also added to the table semi-automatically using the Field Calculator tool.

#### 2.3.3 Analysis of Image Mosaics

**Figure 3:**
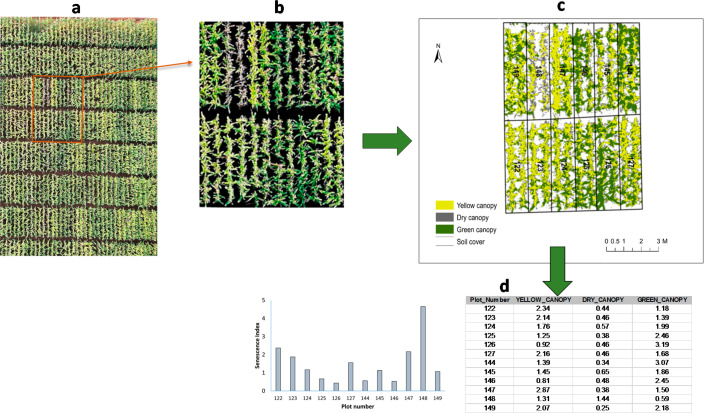
(**a**) Aerial image mosaic of a maize hybrid trials with 150 plots each; (**b**) Preprocessed details of a portion of the field; (**c**) Classification of soil (white) and green canopy (green), yellow canopy (yellow), and dry canopy (gray); (**d**) Results table.

An image enhancement step was conducted using the image analysis tool in ArcGIS 10.1 to improve the visual contrast and sharpness of the crop canopy to enable a better classification. An RGB composite linear stretch implementing the minimum–maximum method was used with minimum threshold values corresponding to the peak of the histogram and the maximum threshold at 255 to increase the spectral difference between the background and canopy pixels (Figure 3a,b). In addition, a gamma correction threshold was set at 0.9. Finally, a sharpen filter was used to allow easier distinction of canopy features. These image enhancement methods were applied to the rendered screen display and not to the mosaic datasets; hence, they did not have impact on the extracted data.

Image classification was performed using Maximum Likelihood Classification (MLC), a pixel-based or supervised classification method in ArcGIS 10.1. Firstly, an image classification scheme consisting of soil cover and yellow, green, and dry canopy was established. Representative training samples that adequately covered all pixel intensities for each class were then manually selected visually on the image. This was achieved by identifying part of the image with increased variability in pixel intensities for each class to be trained. Separation and distribution of the training samples were evaluated using the scatterplot and histogram windows in the training sample manager. After selecting enough training samples for each class, a signature file was generated and saved. The MCL was then performed by using the image and signature as input files to produce a classified image that was used for further plot level data extraction (Figure 3c).

#### 2.3.4 Data Extraction

Plot level data for maize canopy features were extracted from the classified image and quantified as area (m2) per plot which was divided by the plot area to get the canopy relative area. In order to extract these plot level data, the FieldPlot file was first converted to a raster file using the Feature to Raster conversion tool with ObjectID attribute as an input attribute which uniquely identifies each plot. The conversion to raster step ensures consistent results for every plot level extraction, unlike when this is performed on-the-fly by directly using the FieldPlot (a vector file). This raster file was input as zone data and the classified image as class data for extraction of plot level areas using the Tabulate Area tool. A result table of canopy classes was computed for the entire three trials with unique IDs. The attributes of this table were then added to the field plot file using the Join Field tool based on the corresponding unique IDs. The final field plot table (Figure 3d) was then exported to Excel for further data analysis.

### 2.4 Senescence Index

Based on the different color classes and given that any part of a leaf with yellow or brown (dry) color was classified as undergoing or having succumbed to senescence, a senescence index was proposed as the ratio between senesced canopy and the total canopy cover:

$$S_{I}=\left(Y_{C}+D_{C}\right)/\left(Y_{C}+D_{C}+G_{C}\right)$$ (1)

where *G_C_* = green canopy cover, *Y_C_* = yellow canopy cover, and *D_C_* = dry canopy cover.

### 2.5 Ground Truthing

To compare data extracted from the aerial images with a visual assessment, visual senescence scores for each plot were taken the day after the second flight (75 DAS) by trained field technicians. The visual scores were on a scale of 0 to 100 as the percentage of estimated total leaf area that is dead.

### 2.6 Statistical Analysis

Broad-sense heritabilities and genetic correlations were computed using Meta-R(multi-environment trial analysis with R for windows) version 6.01 01 [26] and compared among image variables and reference measurements. Linear models were implemented using REML (restricted maximum likelihood) to calculate BLUEs (best linear unbiased estimations) and BLUPs (best linear unbiased predictions) and estimate the variance components.

The broad-sense heritability of a given trait at an individual environment was calculated as

$$H^{2}=\frac{\sigma_{g}^{2}}{\sigma_{g}^{2}+\sigma_{g}^{2}/nreps}$$ (2)

where σ^2^_g_ and σ^2^_e_ are the genotype and error variance components, respectively, and *nreps* is the number of replicates.

The genetic correlation between traits was calculated as

$$\rho_{g}=\frac{\bar{\sigma_{g\left(jj^{\prime }\right)}}}{\bar{\sigma_{g\left(j\right)}\sigma_{g\left(j^{\prime }\right)}}}$$ (3)

where $\bar{\sigma_{g\left(jj^{\prime }\right)}}$ is the arithmetic mean of all pairwise genotypic covariances between traits *j* and *j*^ʹ^, and $\bar{\sigma_{g\left(j\right)}\sigma_{g\left(j^{\prime }\right)}}$ is the arithmetic average of all pairwise geometric means among the genotypic variance components of the traits.

The relationships between the image variables and reference measurements were tested for significant correlation using the Pearson correlation coefficient.

## 3. Results

### 3.1 Ground Canopy Cover

The data extracted from aerial images exhibited a moderately high heritability for total canopy cover (H^2^ = 0.602) and a significantly positive genetic correlation with grain yield (ρg = 0.792, p < 0.001) (Table 1). Similarly, the three canopy features (yellow, dry, and green canopy areas), as well as the remaining green cover, presented a moderately high heritability (H^2^ > 0.5) with a significantly positive genetic correlation with grain yield, particularly for the yellow and green canopy areas (Table 1). The dry canopy area had the highest heritability but the lowest genetic correlation with grain yield(ρg = −0.301). In addition, all these parameters showed similar or higher heritability as compared to grain yield.

**Table 1:** Broad-sense heritabilities (H^2^) and means for grain yield and canopy features (canopy cover) and genetic correlations (ρg) of these features with grain yield in a maize hybrid evaluated under low soil nitrogen at Harare, Zimbabwe. RGC (remaining green cover) was calculated as the ratio between the green canopy cover and the plot area. (Data are means of 450 plots).

	Canopy			Total Cover	RGC	Grain Yield(Mg ha-1)
	Yellow	Dry	Green			
Heritability	0.526	0.766	0.544	0.602	0.547	0.547
Mean	1.625	0.376	2.379	0.660	0.358	1.670
Genetic correlation (ρg)	0.602 ^**^	−0.301 ^*^	0.616 ^***^	0.792 ^***^	0.650 ^***^	-
n Replicates	3	3	3	3	3	3

* = p < 0.05

** = p < 0.01

*** = p < 0.001.

### 3.2 Leaf Senescence

**Figure 4:**
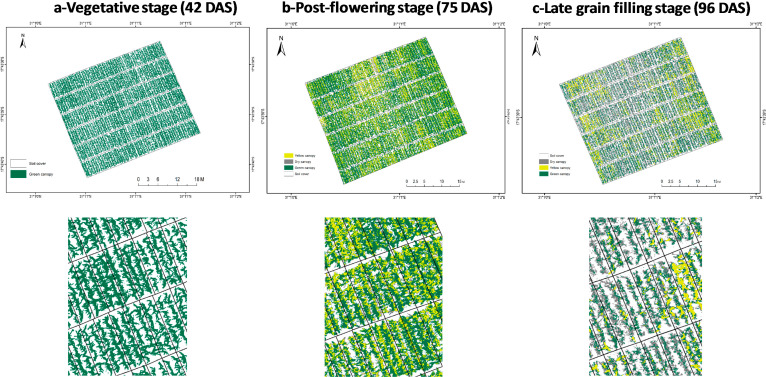
Time sequence aerial images of maize hybrids at three different developmental stages grown at the International Maize and Wheat Improvement Center (CIMMYT)–Harare research station in Zimbabwe. The trials were composed of 50 varieties each, planted using an alpha lattice design with three replicates. (DAS = days after sowing).

This research showed that time sequence data analysis could be conducted using an RGB sensor to monitor crop vigor and senescence patterns (Figure 4). The senescence index derived from aerial imaging data showed a moderately high heritability, similar to that of grain yield with a significant genetic correlation between the two (Tables 1 and 2). On the other hand, the visual assessment data presented a moderately high heritability, except for the first senescence scores (sen1); however, there was no significant genetic correlation with grain yield for the three senescence scores (Table 2).

**Table 2:** Broad-sense heritabilitie (H2) and mean of canopy senescence and its genetic correlation with grain yield in three maize hybrid trials (composed of 50 varieties each) evaluated under low soil nitrogen at Harare, Zimbabwe. (Data are means of 450 plots).

	Aerial Imaging		Visual Assessment	
	Sen. Index	Sen1	Sen2	Sen3
Heritability	0.529	0.285	0.585	0.500
Mean	0.466	12.731	28.666	61.944
Genetic correlation with yield	−0.397 ^**^	−0.179	0.006	−0.101
n Replicates	3	3	3	3

** = p < 0.01, Sen. = canopy senescence. Sen. index (aerial imaging) corresponds to Sen3 (visual assessment).

**Figure 5:**
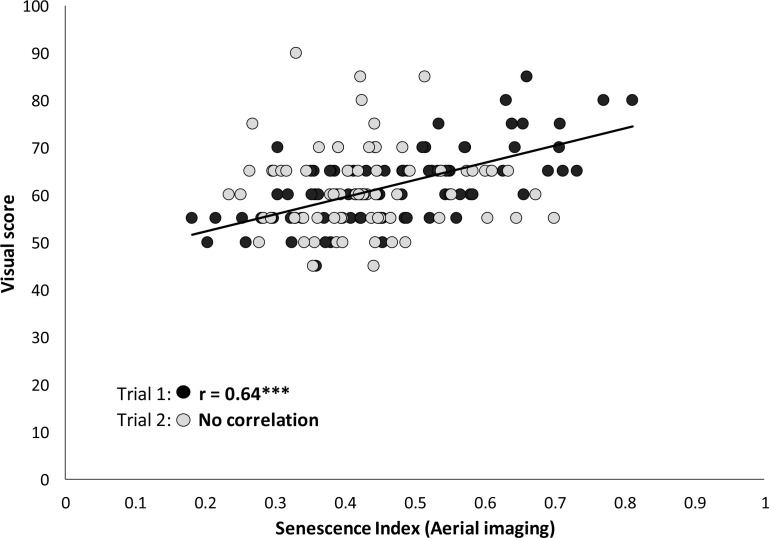
Relationship between visual score and senescence index derived from aerial imaging data for two different field trials.

The comparison between visual assessment of crop senescence and the senescence index derived from aerial imaging pointed out that the correlation between the two is highly variable from one trial to another (Figure 5). There was a significant correlation between the two variables for Trial 1 (r = 0.64, p < 0.001), whilst no correlation was observed for Trial 2 despite the fact that the senescence index data were collected following the same procedure for the two trials.

## 4. Discussion

UAV-based imagery is commonly used in crop mapping for stress detection, due to the high spatial, spectral, and temporal resolutions of the images that are collected [13,21,27]. However, its application in crop improvement is not yet effective, partly due to challenges related to the cost of sensors and small plot level data extraction. This work showed that RGB aerial imaging could be an effective approach for generating crop cover and senescence data at low cost and from plots as small as two rows that are four meters long each. In addition, the data generated using the aerial imaging showed that the different canopy classes had a moderately high heritability and a significant genetic correlation with grain yield (Table 1). The positive genetic correlation between the yellow canopy and grain yield might be an indication of an effective remobilization process. From previous reports, this type of platform has been used in low N stress detection/senescence in crops like corn or wheat [28,29], as well as for estimating final yield. Crop cover can also be estimated using canopy reflectance and surface [30,31]; however, the cost of sensors might be higher as compared to RGB imaging. The image processing methodology used in this work may seem to take a long time, but once the pipeline is established, the data generation can be done quite fast; this is particularly the case if cloud computing is integrated in the pipeline.

Crop cover is often assessed visually or with ground-level digital images. This would require substantial labor, especially in large breeding trials. For trials of several 1000 plots, ground-based estimates by visual scores or with cameras may take many hours/days to collect, compared to the 10 to 15 min flights that are possible with a UAV platform [10]. According to [29], remote sensing could provide inexpensive, large-area estimates of N status in wheat. When compared to the Normalized Difference Vegetation Index (NDVI) at early growth stage, the pixel-based data accuracy tends to be higher [32] because there is no significant soil influence as in the case of NDVI. In sorghum, a strong correlation between crop cover estimated using UAV imagery and plant count was reported [10]. However, in the current work, there was no correlation between the two parameters, most probably due to variations in canopy architecture between the maize hybrids in the trials (data not shown).

Leaf senescence is an indicator of plant age but can also be an important phenotypic trait for the assessment of a plant’s response to stress and is therefore routinely used in selection by plant breeders [25]. Visual assessment of senescence, however, is time consuming, inaccurate, and subjective. The correlation between imaging-based senescence data and visual scores was highly variable (Figure 5) largely because the visual scoring may not be as consistent as required as it depends on the training and subjective appreciations of the staff devoted to that task. The use of imaging technologies and UAVs can provide a high-throughput phenotyping alternative to allow plant breeding programs to undertake quantitative screens of large breeding populations [10,33]. For example, in sorghum, a multispectral sensor was successfully used for leaf senescence monitoring [34]. On the other hand, the digital aerial imaging method can also assist in assessing the stay-green trait using the remaining green area cover,which showed a moderately high heritability and a strong genetic correlation with grain yield (Table 1). This trait could be important to consider, especially under non-limited nitrogen conditions.

## 5. Conclusions

Selection cost reduction is an essential component of the breeding efficiency improvement process. This study has shown the feasibility of using consumer-grade digital cameras onboard low-cost UAVs for the estimation of important plant phenotypic traits like canopy cover and canopy senescence. With advances in image analysis methods, the rapid cost reduction of sensors, and effective image processing software, there is still potential for wider applications of field-based phenotyping by UAVs. The main advantage of UAVs is that they enable the generation of data at the high resolutions needed for accurate crop parameter estimations, and allow in-season dynamic assessment of the crop due to their ability to fly missions at high temporal frequencies. The use of UAV-based technology is projected to grow exponentially in the next couple of years, translating the development and increased availability of robust aerial-sensing-based crop phenotyping methods to plant breeders and the research community at large. This is anticipated to significantly assist in improving selection efficiency through higher precision and accuracy, and the reduced time and cost of data collection.
